# Serum cardiotrophin-1 and interleukin-17 levels are elevated and correlate with disease severity in acne vulgaris

**DOI:** 10.1007/s11845-026-04287-1

**Published:** 2026-02-24

**Authors:** Fatma Sengul-Bag, Fikret Akyurek, Fatma Tuncez-Akyurek, Gülcan Saylam-Kurtipek

**Affiliations:** 1 Department of Biochemistry, Faculty of Pharmacy, University of Adıyaman, Adıyaman, 02040 Türkiye; 2 Department of Medical Biochemistry, Faculty of Medicine, University of Selçuk, Konya, 42250 Türkiye; 3 Department of Dermatology, Faculty of Medicine, University of Selçuk, Konya, 42250 Türkiye

**Keywords:** Acne vulgaris, Cardiotrophin-1, Inflammation, Interleukin-17

## Abstract

**Background:**

Acne vulgaris is a chronic inflammatory disorder of the pilosebaceous unit influenced by innate and adaptive immune responses. Interleukin-17 (IL-17) and cardiotrophin-1, two mediators involved in inflammation and metabolic stress, have been proposed as potential contributors to acne pathophysiology. This study aimed to compare serum IL-17 and cardiotrophin-1 levels between acne patients and healthy controls and to assess their relationship with disease severity.

**Methods:**

In this study, 57 acne patients and 57 healthy controls were enrolled. Serum IL-17 and cardiotrophin-1 levels were measured by ELISA. Acne severity was classified using the Global Acne Grading System. Lipid profiles and hematological parameters were also assessed.

**Results:**

Serum IL-17 and cardiotrophin-1 levels were significantly higher in the acne group compared to controls (*p* < 0.001). Within the patient cohort, the severe acne subgroup had significantly higher levels of both cytokines than the mild acne subgroup. A positive correlation was found between IL-17 and cardiotrophin-1 (r = 0.549, *p* < 0.001), and both were correlated with acne severity (cardiotrophin-1: r = 0.735; IL-17: r = 0.453; *p* < 0.001). ROC analysis demonstrated high diagnostic accuracy for both biomarkers, with an AUC of 0.945 (95%CI:0.909–0.981) for cardiotrophin-1 and 0.904 (95%CI:0.852–0.956) for IL-17. Additionally, leukocyte, neutrophil, and platelet indices, as well as total cholesterol and high-density lipoprotein cholesterol levels, were significantly elevated in patients.

**Conclusions:**

The elevated levels of IL-17 and cardiotrophin-1 and their correlation with disease severity suggest their potential role as inflammatory biomarkers in acne. ROC analysis showed strong discrimination, but acne is clinically diagnosed; biomarkers require multicenter, longitudinal validation.

## Introduction

Acne vulgaris is a chronic, heterogeneous inflammatory disorder of the pilosebaceous unit. Although it is among the most prevalent dermatological diseases during adolescence, it also remains a significant health problem in adulthood [1]. Recent research indicates that acne is associated with complex biological processes that influence the immune response both locally in the cutaneous microenvironment and systemically. As a result, acne is increasingly regarded as an inflammatory disease model shaped by interactions among skin barrier dysfunction, microbial components, and immunomodulation [2, 3].

Its pathogenesis is not limited to the classic inflammatory response triggered solely by *Cutibacterium acnes* colonization; it involves a broader process regulated by innate immune receptors, inflammatory cytokine networks, and adaptive immune cell subsets [4]. In this context, interleukin-17 (IL-17), produced by T helper 17 (Th17) cells, has emerged as a critical mediator in the initiation and progression of acne. Studies have shown that *Cutibacterium acnes* promotes IL-17 synthesis via signaling through Toll-like receptors, and that this cytokine, in turn, enhances keratinocyte proliferation, accelerates neutrophil recruitment, and amplifies the release of antimicrobial peptides [5]. Findings of pronounced activation of the IL-17/Th17 axis in acne lesions are supported by studies reporting elevated IL-17 levels in both skin and serum [6].

Beyond local cutaneous inflammation, acne vulgaris has been increasingly associated with systemic inflammatory and metabolic alterations [7, 8]. In this context, cardiotrophin-1, a multifunctional cytokine in the IL-6 family, is a biologically plausible candidate for investigation [9]. Cardiotrophin-1 is a key regulator of glucose and lipid metabolism and is closely linked to insulin resistance and chronic metabolic stress [10, 11]. Elevated cardiotrophin-1 levels have been reported in systemic inflammatory and metabolic conditions such as obesity, metabolic syndrome, and atherosclerosis, highlighting its role at the intersection of metabolic regulation and immune activation [12]. Given the established associations between acne vulgaris, systemic inflammation, and metabolic dysregulation [7], we hypothesized that cardiotrophin-1 may also be involved in acne pathophysiology. Additionally, as a member of the IL-6 cytokine family, cardiotrophin-1 may indirectly interact with the Th17/IL-17 axis, which is known to play a central role in acne-related inflammation; however, this potential relationship has not yet been explored. Therefore, evaluating serum cytokine profiles that integrate metabolic and inflammatory pathways is essential for elucidating the systemic inflammatory response in acne vulgaris and for identifying biomarkers associated with disease severity. This study aimed to investigate serum IL-17 and cardiotrophin-1 levels in individuals with acne vulgaris, compare them with healthy controls, and assess their correlation with disease severity and with each other.

## Materials and methods

### Study population and design

This case–control study enrolled 57 patients diagnosed with acne vulgaris at our outpatient clinic. The control group consisted of 57 healthy volunteers recruited from individuals presenting for routine health checks at the same center. Controls were matched to the patient group for age and sex distribution and had no diagnosed systemic or dermatological pathology.

The research protocol was approved by the Local Ethics Committee of Selçuk University Faculty of Medicine (approval number: 2025/605). The study was conducted in accordance with the ethical principles of the Declaration of Helsinki.

### Inclusion and exclusion criteria

Eligibility criteria for the study cohort included being over 18 years of age and having a diagnosis of acne vulgaris. Individuals with any dermatological condition other than acne vulgaris were excluded. Additional exclusion criteria were the presence of systemic diseases (e.g., metabolic syndrome, diabetes mellitus, cardiovascular disease, malignancy, rheumatological or autoimmune diseases) and a history of systemic or topical treatment for acne vulgaris within the preceding three months. Individuals who were pregnant or lactating, or who had used immunosuppressive, antibiotic, or anti-inflammatory medication within the last month were also excluded. For the control group, all exclusion criteria applied to the patient group were enforced; additionally, the absence of any active systemic or dermatological disease was confirmed.

### Clinical assessment and acne severity classification

Acne severity was objectively assessed using the validated Global Acne Grading System established by Doshi et al. [13]. This system calculates a total severity score based on the type and density of lesions on the face and upper torso. Based on these total scores, patients were categorized into two groups: a score of 1–30 was classified as “Mild” acne and a score of 31–38 as “Severe” acne.

### Biochemical analyses

Blood samples were collected from all participants during routine clinical examinations. The separated serum was stored at −80 °C until analysis. On the day of the assay, samples were thawed, and serum levels of cardiotrophin-1 and IL-17 were measured using commercially available Enzyme-linked immunosorbent assay (ELISA) kits (Bioassay Technology Laboratory, Catalog No: E1228Hu and E0142Hu, respectively), according to the manufacturers’ instructions. The sandwich ELISA principle was applied for both parameters. Briefly, pre-coated plates were incubated with standards and samples for 60 min at 37 °C. After sequential addition of a biotin-conjugated detection antibody and streptavidin-HRP, unbound reagents were removed by washing. The enzymatic reaction was initiated by adding tetramethylbenzidine substrate solution and incubating for 10 min at 37 °C in the dark. The reaction was terminated by adding an acidic stop solution, and the absorbance of each well was immediately measured at 450 nm using a microplate reader. The concentrations of cardiotrophin-1 (ng/mL) and IL-17 (ng/L) in the samples were interpolated from the standard curve.

Routine biochemical parameters, including glucose, triglycerides, total cholesterol, high-density lipoprotein cholesterol (HDL-C), and low-density lipoprotein cholesterol (LDL-C), were measured photometrically using a Roche Cobas c702 analyzer (USA). Complete blood count parameters were analyzed using a Mindray BC-6000 automated hematology analyzer (Mindray, Shenzhen, China). All measurements were performed according to the manufacturers’ protocols.

### Statistical analysis

Statistical analyses were performed using the R statistical software (version 4.5.0). The normality of continuous variables was assessed using the Shapiro–Wilk test. Normally distributed continuous data are presented as mean ± standard deviation, while non-normally distributed data are presented as median (interquartile range). Categorical variables are expressed as numbers (%). For group comparisons, the Independent Samples t-test was used for normally distributed continuous data, and the Mann–Whitney U test for non-normally distributed data. Categorical variables were compared using the Chi-square test. In addition to p-values, effect size measures were calculated to quantify the magnitude of between-group differences. Cliff’s Delta was used for nonparametric continuous variables, Cohen’s d was applied to parametric continuous variables, and Cramér’s V was calculated for categorical variables. The relationship between cardiotrophin-1 and IL-17 levels and other clinical parameters was analyzed using Spearman’s correlation coefficient. The diagnostic performance of cardiotrophin-1 and IL-17 in distinguishing acne patients from controls and in differentiating disease severity was evaluated using Receiver Operating Characteristic (ROC) curve analysis, and Area Under the Curve (AUC) values were calculated. A p-value < 0.05 was considered statistically significant.

## Results

The study included 57 acne patients and 57 healthy controls. The patient and control groups had similar demographic profiles, with no statistically significant differences in age, body mass index, or sex distribution (*p* > 0.05 for all parameters), as shown in Table 1.

**Table 1:** Comparison of demographic, clinical, and laboratory characteristics between patients with acne vulgaris and healthy controls

Variable	Acne vulgaris (*n* = 57)	Control (*n* = 57)	p-value	Effect size
Age, years	19 (18–20)	18 (18–22)	0.878^1^	−0.016
Body mass index, kg/m^2^	21.4 (19.5–23.5)	21.4 (19.6–24.3)	0.638^1^	−0.051
Sex (Male/Female)	13 (22.8%)/44 (77.2%)	13 (22.8%)/44 (77.2%)	1.000^2^	0.000
Cardiotrophin-1, ng/mL	75.18 (64.37–103.94)	43.82 (33.7–58.48)	< 0.001^1^	0.890
IL-17, ng/L	45.63 (40.00–51.52)	33.36 (26.27–40.19)	< 0.001^1^	0.809
Glucose, mg/dL	91.30 ± 7.15	87.63 ± 6.38	0.005^3^	0.541
Triglycerides, mg/dL	76 (61–100)	68.00 (58–84)	0.058^1^	0.207
Total cholesterol, mg/dL	152.04 ± 26.77	138.54 ± 18.06	0.002^3^	0.591
LDL-C, mg/dL	80.58 ± 20.87	81.55 ± 14.98	0.777^3^	−0.053
HDL-C, mg/dL	59 (51–65)	48 (45–54)	< 0.001^1^	0.506
White Blood Cell Count, K/µL	7.19 (6.14–8.38)	6.09 (5.12–7.34)	< 0.001^1^	0.385
Hemoglobin, g/dL	13.8 (13.2–14.6)	13.7 (13–14.7)	0.986^1^	0.002
Hematocrit, %	41.5 (39.9–43.8)	41.2 (38.9–44)	0.539^1^	0.067
Platelet Count, K/µL	300.16 ± 56.99	279.58 ± 53.35	0.049^3^	0.373
Red Cell Distribution Width, %	13.4 (13–14.2)	13.2 (12.8–13.9)	0.069^1^	0.197
Platelet Distribution Width, fL	12.9 (11.4–14.2)	12.2 (10.7–13.6)	0.044^1^	0.219
Mean Platelet Volume, fL	10.5 (9.8–11.4)	10.2 (9.5–11)	0.112^1^	0.173
Neutrophil Count, K/µL	4.32 (3.45–5.42)	3.5 (2.83–4.38)	< 0.001^1^	0.391
Lymphocyte Count, K/µL	2.32 (2–2.81)	2.17 (1.87–2.48)	0.058^1^	0.206
Monocyte Count, K/µL	0.43 (0.38–0.53)	0.46 (0.37–0.54)	0.836^1^	−0.023
Disease duration, months	40 (30–48)	NA		
Acne severity score	29 (18–35)	NA		

^1^Mann–Whitney U test, ^2^ Chi-square test with Yates continuity correction, ^3^ Independent Samples T-Test

Categorical variables are presented as numbers and percentages [n (%)], while continuous variables are expressed as mean ± standard deviation, median (1st quartile–3rd quartile) or count (n) and percentage (%), as appropriate. Effect sizes were expressed as Cliff’s Delta for non-parametric continuous variables, Cohen’s d for parametric continuous variables, and Cramér’s V for categorical variables. *p*-value < 0.05 was considered as statistically significant. IL-17, interleukin-17; LDL-C, low-density lipoprotein cholesterol; HDL-C, high-density lipoprotein cholesterol; NA, not applicable

**Fig. 1:**
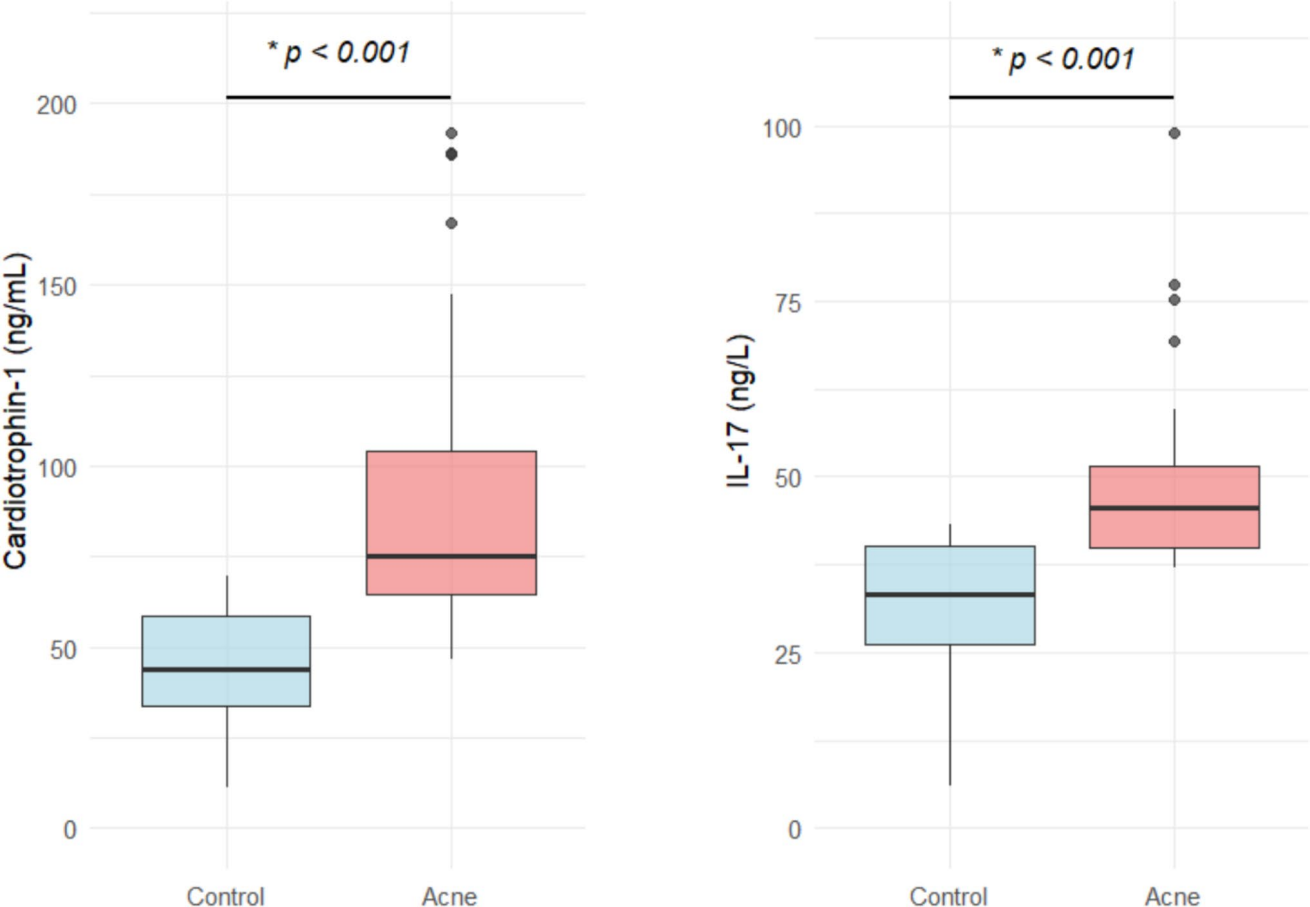
Comparison of serum cardiotrophin-1 and IL-17 levels between acne patients and healthy controls. *p < 0.001 (Mann–Whitney U test)

The acne patient group exhibited significantly elevated serum levels of both cardiotrophin-1 and IL-17 compared to the healthy control group (*p* < 0.001 for both, Table 1, Fig. 1).

**Fig. 2:**
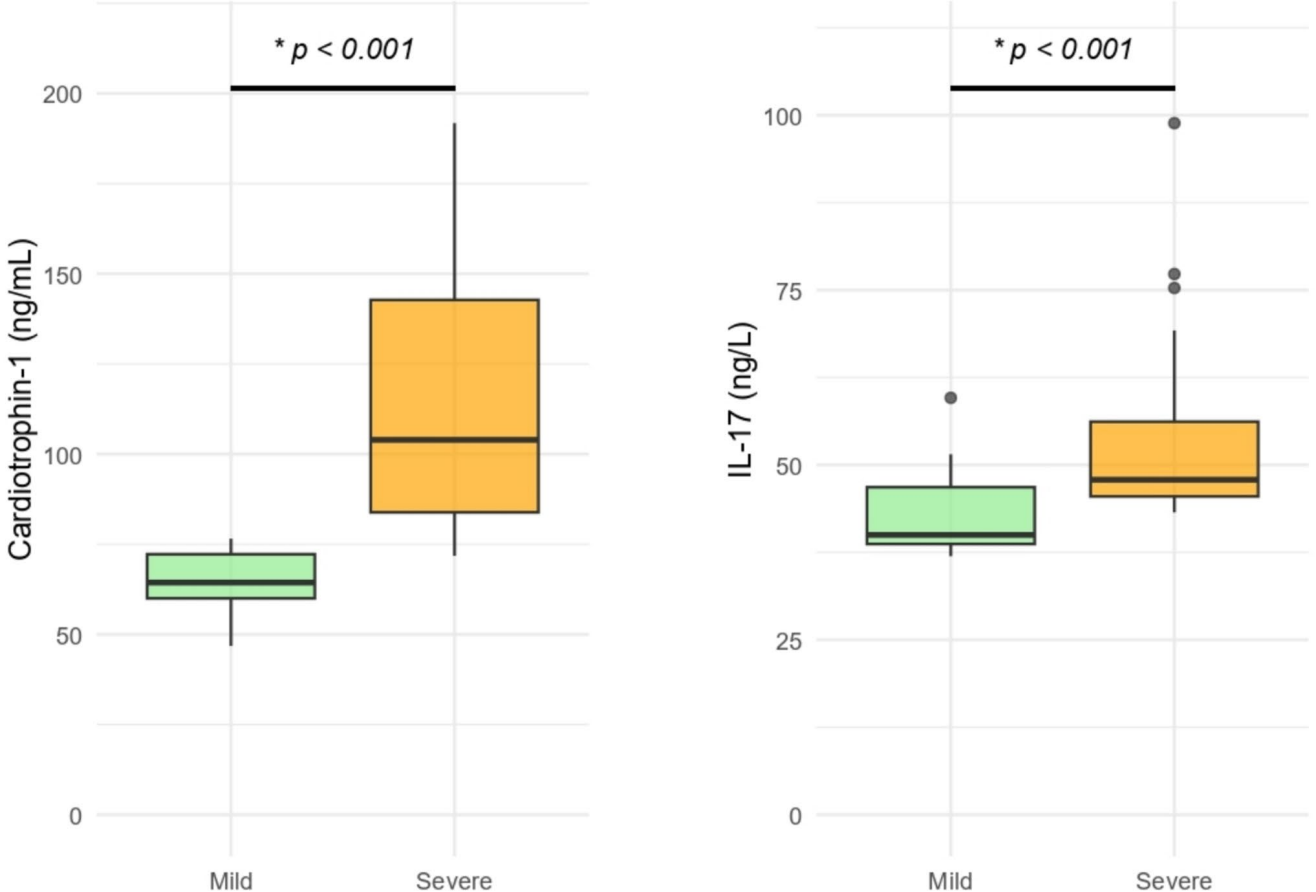
Comparison of serum cardiotrophin-1 and IL-17 levels between mild and severe acne patient subgroups. *p < 0.001 (Mann–Whitney U test)

When the acne patients were stratified into two subgroups based on the Global Acne Grading System—mild (*n* = 29) and severe (*n* = 28)—the patients in the severe acne group demonstrated significantly higher serum levels of both cardiotrophin-1 and IL-17 compared to those in the mild acne group (*p* < 0.001 for both comparisons) (Table 2, Fig. 2).

**Table 2:** Comparison of patients based on acne severity

Variable	Acne-mild (*n* = 29)	Acne-severe (*n* = 28)	*p*-value	Effect size
Age, years	19 (18–21)	19 (18–20)	0.644^1^	−0.069
Body mass index, kg/m^2^	21.3 (19.1–23.3)	21.45 (19.6–23.85)	0.367^1^	0.140
Sex (Male/Female)	6 (20.7%)/23 (79.3%)	7 (25%)/21 (75%)	0.943^2^	0.010
Cardiotrophin-1, ng/mL	64.37 (60–72.23)	103.94 (83.85–142.75)	< 0.001^1^	0.966
IL-17, ng/L	40 (38.7–46.82)	47.88 (45.5–56.19)	< 0.001^1^	0.666
Glucose, mg/dL	90.66 ± 7.16	91.96 ± 7.2	0.494^3^	0.182
Triglycerides, mg/dL	76 (62–90)	78.5 (58–108)	0.861^1^	0.028
Total cholesterol, mg/dL	149.31 ± 21.67	154.86 ± 31.34	0.442^3^	0.207
LDL-C, mg/dL	82.22 ± 20.23	78.87 ± 21.75	0.549^3^	−0.160
HDL-C, mg/dL	55 (48–65)	60.5 (54.75–63.5)	0.434^1^	0.223
White Blood Cell Count, K/µL	6.58 (6.04–8.16)	7.53 (6.75–9.72)	0.061^1^	0.291
Hemoglobin, g/dL	13.8 (12.9–14.6)	13.8 (13.2–14.48)	0.829^1^	0.035
Hematocrit, %	41.1 (39.5–43.8)	41.5 (40.8–43.08)	0.482^1^	0.110
Platelet Count, K/µL	292.38 ± 50	308.21 ± 63.33	0.301^3^	0.278
Red Cell Distribution Width, %	13.4 (12.9–14.8)	13.35 (13.07–14.12)	0.848^1^	0.031
Platelet Distribution Width, fL	13.6 (12.4–14.5)	12.1 (11.15–13.58)	0.086^1^	−0.266
Mean Platelet Volume, fL	10.9 (10.1–11.7)	10.25 (9.6–11)	0.144^1^	−0.227
Neutrophil Count, K/µL	3.75 (3.27–4.79)	4.54 (3.76–6.92)	0.045^1^	0.310
Lymphocyte Count, K/µL	2.35 (2.11–2.67)	2.13 (1.97–2.84)	0.708^1^	−0.059
Monocyte Count, K/µL	0.43 (0.38–0.5)	0.42 (0.38–0.54)	0.767^1^	0.047
Disease duration, months	40 (24–54)	38 (36–48)	0.778^1^	0.043
Acne severity score	18 (15–22)	35 (31–38)	< 0.001^1^	1.000

^1^Mann–Whitney U test, ^2^ Chi-square test with Yates continuity correction, ^3^ Independent Samples T-Test

Categorical variables are presented as numbers and percentages [n (%)], while continuous variables are expressed as mean ± standard deviation, median (1st quartile–3rd quartile) or count (n) and percentage (%), as appropriate. Effect sizes were expressed as Cliff’s Delta for non-parametric continuous variables, Cohen’s d for parametric continuous variables, and Cramér’s V for categorical variables. *p*-value < 0.05 was considered as statistically significant. IL-17, interleukin-17; LDL-C, low-density lipoprotein cholesterol; HDL-C, high-density lipoprotein cholesterol; NA, not applicable

Correlation analysis revealed a positive, moderate correlation between serum cardiotrophin-1 and IL-17 levels (r = 0.549, *p* < 0.001). Furthermore, both biomarkers demonstrated significant positive correlations with the acne severity score (cardiotrophin-1: r = 0.735, *p* < 0.001; IL-17: r = 0.453, *p* < 0.001) (Table 3).

**Table 3:** Correlations of cardiotrophin-1 and ıl-17 levels with clinical and laboratory parameters in patients with acne vulgaris

	Cardiotrophin-1 (ng/mL)		IL-17 (ng/L)	
	Spearman’s *rho*	*p*-value	Spearman’s *rho*	*p*-value
IL-17, ng/L	0.549	** < 0.001**	-	-
Cardiotrophin-1, ng/mL	-	-	0.549	** < 0.001**
Acne severity score	0.735	** < 0.001**	0.453	** < 0.001**
Disease duration, months	0.129	0.339	−0.066	0.624
White Blood Cell Count, K/µL	0.168	0.212	0.051	0.705
Hemoglobin, g/dL	0.038	0.78	0.216	0.106
Hematocrit, %	0.092	0.495	0.269	**0.043**
Platelet Count, K/µL	0.0.138	0.307	−0.194	0.148
Red Cell Distribution Width, %	0.034	0.802	−0.21	0.116
Platelet Distribution Width, fL	−0.272	**0.041**	−0.057	0.672
Mean Platelet Volume, fL	−0.253	0.057	−0.032	0.815
Neutrophil Count, K/µL	0.119	0.378	0.074	0.582
Lymphocyte Count, K/µL	0.037	0.787	−0.095	0.481
Monocyte Count, K/µL	0.133	0.324	−0.154	0.253
Glucose (mg/dL)	0.054	0.69	0.071	0.601
Triglycerides (mg/dL)	0.042	0.754	−0.076	0.576
Total cholesterol (mg/dL)	0.055	0.686	0.095	0.483
LDL-C (mg/dL)	−0.073	0.589	0.033	0.807
HDL-C (mg/dL)	0.014	0.919	−0.16	0.235

Significant relationships denoted as bold. IL-17, interleukin-17; LDL-C, low-density lipoprotein cholesterol; HDL-C, high-density lipoprotein cholesterol

**Fig. 3:**
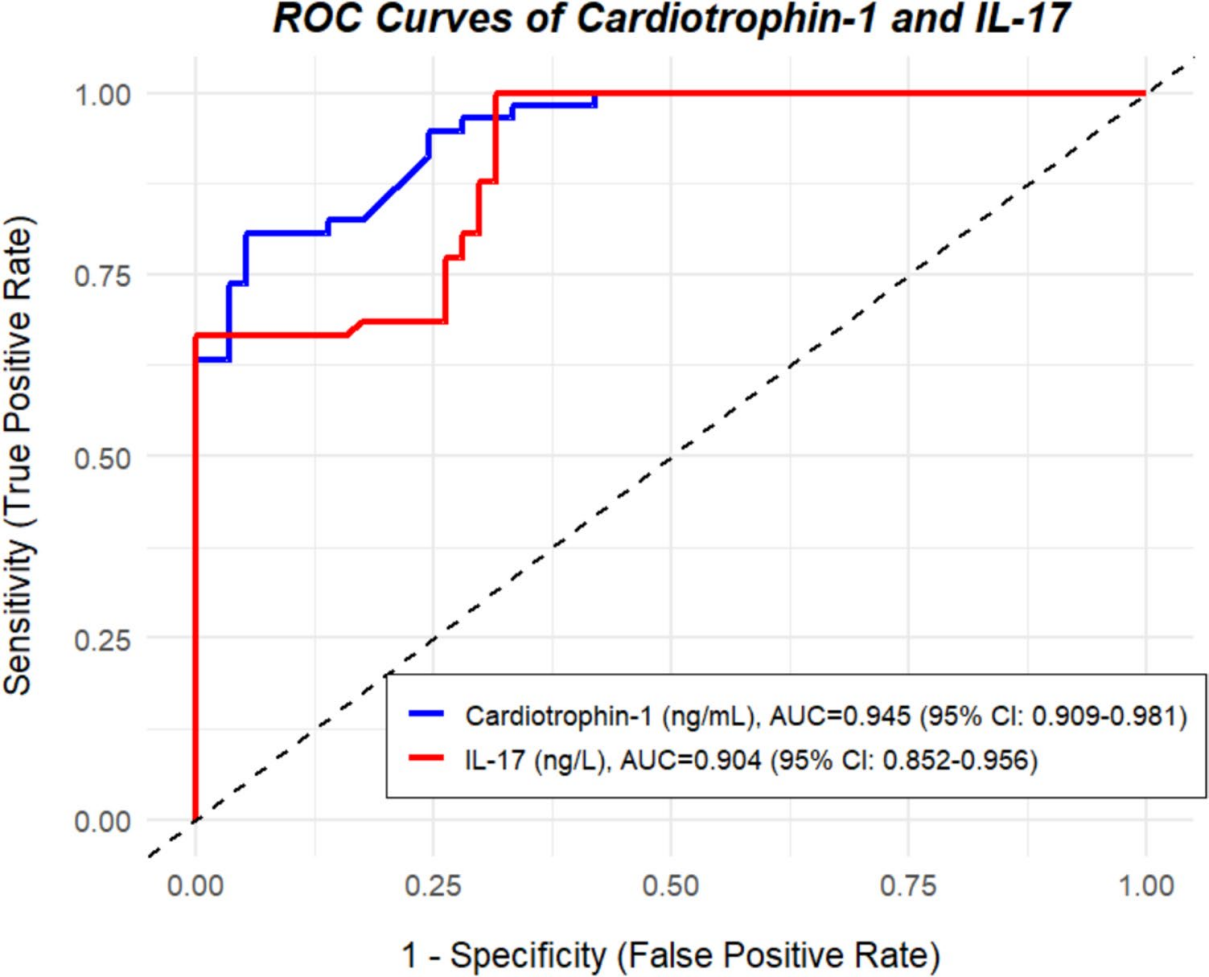
Diagnostic performance of serum cardiotrophin-1 and IL-17 in distinguishing acne patients from healthy controls

ROC curve analysis showed that both cardiotrophin-1 and IL-17 had high diagnostic performance in distinguishing acne patients from healthy controls. The AUC for cardiotrophin-1 was 0.945 (95% CI: 0.909–0.981, *p* < 0.001), and for IL-17, it was 0.904 (95% CI: 0.852–0.956, *p* < 0.001). A cut-off value of ≥ 62.94 ng/mL for cardiotrophin-1 yielded 80.7% sensitivity, 94.7% specificity, 93.9% positive predictive value (PPV), and 83.1% negative predictive value (NPV). For IL-17, a cut-off value of ≥ 36.89 ng/L provided 100% sensitivity, 68.4% specificity, 76% PPV, and 100% NPV (Fig. 3).

## Discussion

This study shows that serum levels of cardiotrophin-1 and IL-17 are significantly higher in individuals with acne vulgaris than in healthy controls and are positively correlated with disease severity. The positive correlation between cardiotrophin-1 and IL-17 suggests that these molecules may participate in overlapping inflammatory pathways. Additionally, ROC curve analysis showed that both biomarkers demonstrated strong statistical discriminatory performance between acne patients and healthy individuals.

The pivotal role of immune responses triggered by *Cutibacterium acnes* in acne pathogenesis is well established. *Cutibacterium acnes* enhances inflammatory responses in keratinocytes and promotes activation of the Th17/IL-17 axis through Toll-like receptor–mediated signaling, leading to increased keratinocyte proliferation, neutrophil recruitment, and antimicrobial peptide release [4, 14, 15]. Elevated serum IL-17 levels observed in the present study likely reflect a systemic Th17 response and are consistent with previous reports demonstrating activation of the IL-17/Th17 axis in both early and established acne lesions [6, 16]. IL-17 contributes to inflammation through induction of multiple cytokines and chemokines, mobilization of neutrophils, and activation of various immune and non-immune cell types, including keratinocytes and fibroblasts [17–19]. In addition, evidence that innate immune cells such as mast cells can also produce IL-17 suggests that the source of this cytokine in acne is not limited to Th17 cells [20].

Cardiotrophin-1 is a pleiotropic molecule involved in metabolic homeostasis, cardiovascular function, and immune regulation [9]. Previous studies have reported elevated cardiotrophin-1 levels in conditions characterized by systemic biological stress, such as obesity, metabolic syndrome, insulin resistance, and chronic inflammation [10, 11]. Given evidence suggesting an association between acne vulgaris and systemic inflammation [20], our finding of increased cardiotrophin-1 levels is consistent with these prior observations.

The positive correlation between cardiotrophin-1 and IL-17 suggests that these two mediators may be involved in shared signaling pathways within inflammatory processes. The biological plausibility of this relationship is supported by cardiotrophin-1's membership in the IL-6 family, as IL-6 is a key cytokine known to drive Th17 cell differentiation [21]. Furthermore, the strong correlation between cardiotrophin-1 and acne severity (r = 0.735) implies that it may serve as a biological indicator of disease activity. Similarly, the moderate positive correlation of IL-17 with acne severity (r = 0.453) indicates that this cytokine is associated not only with mean group differences but may also be quantitatively linked to disease progression.

The hematological findings in our study also align with IL-17-driven inflammatory mechanisms. The significantly elevated white blood cell and neutrophil counts in the acne group are consistent with literature demonstrating IL-17’s role in promoting neutrophil mobilization. Increased platelet count and platelet distribution width may reflect low-grade inflammatory activity manifesting in systemic blood parameters [22]. The concurrence of these hematological changes with elevated cardiotrophin-1 and IL-17 supports the idea that acne may have a mild systemic inflammatory component, extending beyond a purely localized condition. Regarding metabolic parameters, total cholesterol and HDL-C levels were significantly higher in patients with acne vulgaris compared to healthy controls, while triglyceride and LDL-C levels did not differ significantly between the groups. These findings suggest that metabolic alterations in acne vulgaris may be selective rather than indicative of a generalized dyslipidemic profile [23]. Elevated total cholesterol may be related to increased steroidogenesis and sebum production, while higher HDL-C levels could represent a compensatory response to systemic inflammatory activity. The absence of significant differences in triglyceride and LDL-C levels underscores the need for cautious interpretation of metabolic involvement in acne vulgaris. The lack of a significant correlation between disease duration and cardiotrophin-1 or IL-17 levels suggests that the elevation of these cytokines may reflect active inflammatory processes rather than disease chronicity. This supports the idea that IL-17 and cardiotrophin-1 may function more as biochemical indicators of current inflammatory activity.

Our ROC analysis showed that both IL-17 and cardiotrophin-1 demonstrated strong statistical discriminatory performance in distinguishing patients with acne vulgaris from healthy controls. The observed performance of IL-17 aligns with previous studies reporting its association with inflammatory acne phenotype [6]. Similarly, the high AUC value for cardiotrophin-1 highlights this biomolecule as a novel and promising candidate biomarker that warrants further investigation in acne vulgaris. The high positive predictive value of cardiotrophin-1 (93.9%) and the high negative predictive value of IL-17 (100%) observed in the ROC analysis indicate robust statistical classification performance; however, these findings should be interpreted with caution.

It is important to emphasize that acne vulgaris is primarily a clinically diagnosed condition, and the present ROC analysis reflects statistical discriminatory capacity rather than proposing clinically applicable diagnostic cut-off values. Therefore, serum IL-17 and cardiotrophin-1 levels are not intended to replace clinical assessment but may serve as supportive biomarkers in research settings or in evaluating disease severity. Since current acne assessment largely relies on subjective grading systems, quantitative biomarkers such as IL-17 and cardiotrophin-1 may, after further validation, contribute to a more objective characterization of inflammatory burden or disease severity. Nonetheless, the clinical applicability of these biomarkers must be confirmed through larger, multicenter, and longitudinal studies across diverse populations.

Strengths of this study include the classification of acne patients using the validated Global Acne Grading System and the concurrent evaluation of two cytokines, IL-17 and cardiotrophin-1, which represent distinct biological axes, within the same cohort. Additionally, the investigation of their diagnostic performance via ROC curve analysis provides an objective and significant contribution to the interpretation of our findings. A primary limitation is the single-center and cross-sectional design, which may limit generalizability and precludes determination of causal relationships between fluctuations in IL-17/cardiotrophin-1 levels and acne pathogenesis. Furthermore, the lack of tissue-level cytokine expression analysis creates uncertainty regarding which specific components of the cutaneous inflammatory process are reflected by the observed serum cytokine levels and their associated hematological and metabolic changes. Moreover, serum cytokine concentrations may not fully reflect local cutaneous cytokine activity within acne lesions, and the absence of paired tissue or lesional measurements limits inferences about the relationship between systemic biomarkers and the local inflammatory microenvironment.

In conclusion, this study demonstrates that serum levels of IL-17 and cardiotrophin-1 are elevated in individuals with acne vulgaris and correlate with disease severity. ROC analysis indicates that both biomarkers show strong statistical discriminatory performance between acne patients and healthy controls. The concurrent observation of an inflammatory shift in hematological parameters alongside the increase in IL-17 and cardiotrophin-1 supports the hypothesis that acne may be associated with a low-grade systemic inflammatory response.

Future studies should focus on elucidating the precise role of cardiotrophin-1 within the pilosebaceous unit. Investigating the utility of IL-17 and cardiotrophin-1 levels in predicting disease course or treatment response would be valuable. Additionally, correlating tissue expression of these cytokines with their serum levels will provide a clearer understanding of the biological significance of these systemic biomarkers. Larger-scale, prospective, and mechanistic studies are warranted to comprehensively define the role of IL-17 and cardiotrophin-1 in the pathogenesis of acne vulgaris and their potential clinical applications.

## Data Availability

Data are available from the corresponding author upon reasonable request.

## References

[1] Lynn DD, Umari T, Dunnick CA, Dellavalle RP (2016) The epidemiology of acne vulgaris in late adolescence. Adolesc Health Med Ther 7:13–25. 10.2147/ahmt.S55832 26955297 10.2147/AHMT.S55832 PMC4769025

[2] Cong T-X, Hao D, Wen X et al (2019) From pathogenesis of acne vulgaris to anti-acne agents. Arch Dermatol Res 311:337–349. 10.1007/s00403-019-01908-x 30859308 10.1007/s00403-019-01908-x

[3] Vasam M, Korutla S, Bohara RA (2023) Acne vulgaris: a review of the pathophysiology, treatment, and recent nanotechnology based advances. Biochem Biophys Rep 36:101578. 10.1016/j.bbrep.2023.101578 38076662 10.1016/j.bbrep.2023.101578 PMC10709101

[4] Kistowska M, Meier B, Proust T et al (2015) *Propionibacterium acnes* promotes Th17 and Th17/Th1 responses in acne patients. J Invest Dermatol 135:110–118. 10.1038/jid.2014.290 25010142 10.1038/jid.2014.290

[5] Agak GW, Kao S, Ouyang K et al (2018) Phenotype and antimicrobial activity of Th17 cells induced by *Propionibacterium acnes* strains associated with healthy and acne skin. J Invest Dermatol 138:316–324. 10.1016/j.jid.2017.07.842 28864077 10.1016/j.jid.2017.07.842 PMC5794628

[6] Kelhälä HL, Palatsi R, Fyhrquist N et al (2014) IL-17/Th17 pathway is activated in acne lesions. PLoS ONE 9:e105238. 10.1371/journal.pone.0105238 25153527 10.1371/journal.pone.0105238 PMC4143215

[7] Endres LM, Bungau AF, Tit DM et al (2025) Acne vulgaris associated with metabolic syndrome: a three-case series highlighting pathophysiological links and therapeutic challenges. Diagnostics (Basel) 15:2018. 10.3390/diagnostics15162018 40870870 10.3390/diagnostics15162018 PMC12386121

[8] Mohammed GF, Al-Dhubaibi MS, Bahaj SS et al (2024) Acne vulgaris: a warning sign for diagnosing metabolic syndrome. Arch Dermatol Res 317:5. 10.1007/s00403-024-03495-y 39520560 10.1007/s00403-024-03495-y

[9] Watanabe T, Konii H, Sato K (2018) Emerging roles of cardiotrophin-1 in the pathogenesis and biomarker of atherosclerosis. J 1:94–105. 10.3390/j1010010

[10] Moreno-Aliaga MJ, Pérez-Echarri N, Marcos-Gómez B et al (2011) Cardiotrophin-1 is a key regulator of glucose and lipid metabolism. Cell Metab 14:242–253. 10.1016/j.cmet.2011.05.013 21803294 10.1016/j.cmet.2011.05.013

[11] López-Yoldi M, Moreno-Aliaga MJ, Bustos M (2015) Cardiotrophin-1: a multifaceted cytokine. Cytokine Growth Factor Rev 26:523–532. 10.1016/j.cytogfr.2015.07.009 26188636 10.1016/j.cytogfr.2015.07.009

[12] Escoté X, Gómez-Zorita S, López-Yoldi M et al (2017) Role of omentin, vaspin, cardiotrophin-1, TWEAK and NOV/CCN3 in obesity and diabetes development. Int J Mol Sci 18:1770. 10.3390/ijms18081770 28809783 10.3390/ijms18081770 PMC5578159

[13] Doshi A, Zaheer A, Stiller MJ (1997) A comparison of current acne grading systems and proposal of a novel system. Int J Dermatol 36:416–418. 10.1046/j.1365-4362.1997.00099.x 9248884 10.1046/j.1365-4362.1997.00099.x

[14] Sanford JA, Gallo RL (2013) Functions of the skin microbiota in health and disease. Semin Immunol 25:370–377. 10.1016/j.smim.2013.09.005 24268438 10.1016/j.smim.2013.09.005 PMC4219649

[15] Suh DH, Kwon HH (2015) What’s new in the physiopathology of acne? Br J Dermatol 172(Suppl 1):13–19. 10.1111/bjd.13634 25645151 10.1111/bjd.13634

[16] Ebrahim AA, Mustafa AI, El-Abd AM (2019) Serum interleukin-17 as a novel biomarker in patients with acne vulgaris. J Cosmet Dermatol 18:1975–1979. 10.1111/jocd.12934 30964235 10.1111/jocd.12934

[17] Di Cesare A, Di Meglio P, Nestle FO (2009) The IL-23/Th17 axis in the immunopathogenesis of psoriasis. J Invest Dermatol 129:1339–1350. 10.1038/jid.2009.59 19322214 10.1038/jid.2009.59

[18] Weaver CT, Hatton RD, Mangan PR, Harrington LE (2007) IL-17 family cytokines and the expanding diversity of effector T cell lineages. Annu Rev Immunol 25:821–852. 10.1146/annurev.immunol.25.022106.141557 17201677 10.1146/annurev.immunol.25.022106.141557

[19] Rodero MP, Hodgson SS, Hollier B et al (2013) Reduced Il17a expression distinguishes a Ly6c(lo)MHCII(hi) macrophage population promoting wound healing. J Invest Dermatol 133:783–792. 10.1038/jid.2012.368 23235530 10.1038/jid.2012.368

[20] Zouboulis CC, Jourdan E, Picardo M (2014) Acne is an inflammatory disease and alterations of sebum composition initiate acne lesions. J Eur Acad Dermatol Venereol 28:527–532. 10.1111/jdv.12298 24134468 10.1111/jdv.12298

[21] Korn T, Bettelli E, Oukka M, Kuchroo VK (2009) IL-17 and Th17 cells. Annu Rev Immunol 27:485–517. 10.1146/annurev.immunol.021908.132710 19132915 10.1146/annurev.immunol.021908.132710

[22] Akamatsu H, Horio T, Hattori K (2003) Increased hydrogen peroxide generation by neutrophils from patients with acne inflammation. Int J Dermatol 42:366–369. 10.1046/j.1365-4362.2003.01540.x 12755973 10.1046/j.1365-4362.2003.01540.x

[23] Mohammed GF, Al-Dhubaibi MS, Bahaj SS, AbdElneam AI (2023) Alterations in lipid and hormonal titers in patients with acne and their relationship with severity: a case-control study. Health Sci Rep 6:e1322. 10.1002/hsr2.1322 37275673 10.1002/hsr2.1322 PMC10234112

